# Identifying anticancer peptides by using improved hybrid compositions

**DOI:** 10.1038/srep33910

**Published:** 2016-09-27

**Authors:** Feng-Min Li, Xiao-Qian Wang

**Affiliations:** 1 College of Science, Inner Mongolia Agricultural University, Hohhot, 010018, China

## Abstract

Cancer is one of the main causes of threats to human life. Identification of anticancer peptides is important for developing effective anticancer drugs. In this paper, we developed an improved predictor to identify the anticancer peptides. The amino acid composition (AAC), the average chemical shifts (acACS) and the reduced amino acid composition (RAAC) were selected to predict the anticancer peptides by using the support vector machine (SVM). The overall prediction accuracy reaches to 93.61% in jackknife test. The results indicated that the combined parameter was helpful to the prediction for anticancer peptides.

Cancer is the leading cause of death in economically developed countries^1^. The traditional methods for the treatment of cancer are surgical operation, radiotherapy and chemotherapy. However, radiotherapy and chemotherapy are expensive and cause side effect. Since Swedish scientist Boman first found and determined the antimicrobial peptides primary structure of Hyalophora cecropia in 1972, many studies had shown that the antimicrobial peptides have anti tumor activity^2^,^3^. The antimicrobial peptides which have anti tumor activity are called anticancer peptides (ACPs). Anticancer peptides contain 12–50 amino acid residues. Some of these peptides present in membranes with α-helical or β-sheet structures, and the others have particular folds. The mechanism of the action of anticancer peptides includes: necrosis by the cell membrane lytic effect and non-membranolytic^4^,^5^,^6^. Advances in experimental technology have enabled to identify whether a protein belongs to anticancer peptide. However, experimental determination of the anticancer peptide remains time-consuming and laborious. Hence, developing and improving a fast and effective way to predict whether a protein is anticancer peptide would be very necessary. Recently, many methods were focused on the prediction of antimicrobial peptides from primary protein sequences^7^,^8^,^9^. However, the identification of anticancer peptides from primary protein sequences is still at the infant stage. Hajisharifi *et al*.^10^ attempted to identify the anticancer peptide based on pseudo amino acid compositions (PseAAC) and the local alignment kernel by using of the Support Vector Machine (SVM). The prediction was made by the 5-fold cross-validation test and the overall success rate was 89.7%. Recently, Chen *et al*.^11^ predicted the anticancer peptide with the iACP, the better predictive results were obtained.

In this paper, the amino acid composition (AAC), the average chemical shifts (acACS) and the reduced amino acid composition (RAAC) were selected to predict the anticancer peptides with the same datasets as investigated by Hajisharifi *et al*.^10^. The overall prediction accuracy in jackknife test was 93.61% by using the combined parameter AAC + RAAC + acACS for support vector machine (SVM). The predictive results showed significant improvement compared with Hajisharifi’s method.

## Results

### The prediction of anticancer peptides

In order to predict the anticancer peptides, it is very important to choose a classifier and a set of reasonable information parameters from protein sequence. In this paper, the local amino acids composition (AAC), the average chemical shift (acACS) and the reduced amino acid composition (RAAC) were selected to predict the anticancer peptides.

**Figure 1:**
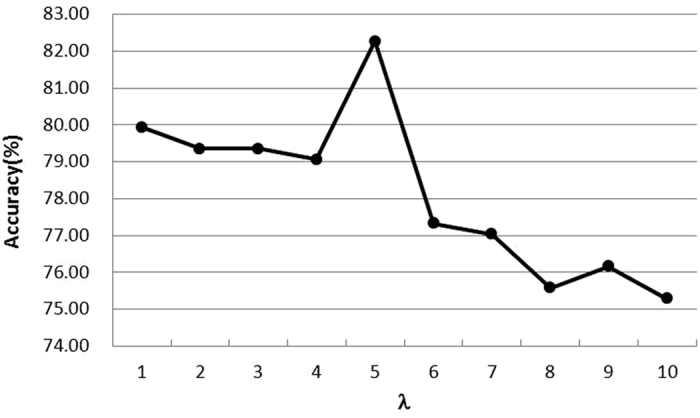
prediction results with respect to the correlation factor *λ* of the acACS based on the jackknife test. The triangle indicates the best results obtained with *λ* = 5.

**Figure 2:**
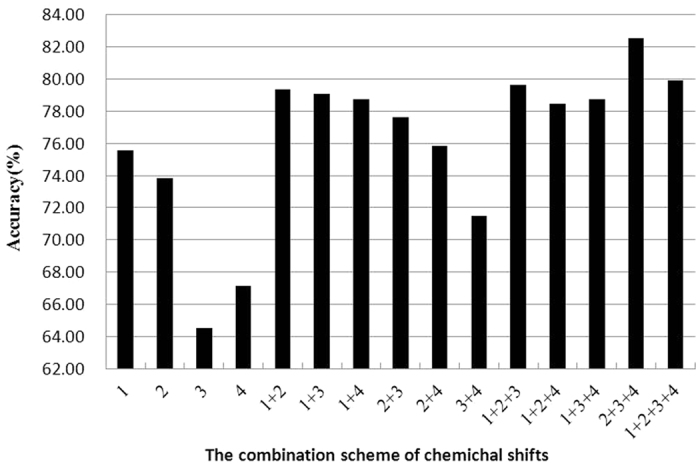
Prediction results of different combination schemes of chemical shifts. Numbers denote the chemical shifts of atoms: 1 for ^1^H_*α*_, 2 for ^1^H_N_, 3 for ^15^N, 4 for ^13^*C*_*α*_.

The acACS vectors were formed based on protein sequence, and then the best *λ* and *i* were selected. In order to obtain the best performance of predicting anticancer peptides, the combined scheme of chemically shifted atoms and the best *λ* were optimized with the maximum accuracy. Results in Fig. 1 showed that the accuracy was the best when *λ* = 5 and in Fig. 2 showed that the prediction result was the best when the combination mode of chemically shifted atoms was 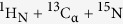. Therefore, the combination mode chemically shifted 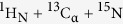 was selected and the correlation factor *λ* was set to 5 for generating the acACS feature vectors.

For facilitating comparison, the benchmark dataset (see Equation (5)) generated by Hajisharifi *et al*.^10^ was employed. The predictive results of anticancer peptides based on AAC, acACS, RAAC, AAC + RAAC, AAC + acACS, RAAC + acACS and AAC + RAAC + acACS by using SVM with jackknife test were recorded in Table 1. The results showed that the combined parameter of AAC + RAAC + acACS was better than other parameters. The overall predictive accuracy (*Q*_*A*_) and Matthew’s correlation coefficient (*MCC*) in jackknife test were 93.61% and 0.867 with the combined parameter of AAC + RAAC + acACS by using of the SVM, respectively. The results indicated that the combined parameter was helpful to the prediction for anticancer peptides.

In order to estimate the effectiveness of the new prediction method, an independent dataset (see Equation (6)) generated by Chen *et al*.^11^ was employed. The independent dataset is not absolutely needed for validating a predictor via the jackknife or K-fold cross-validation, since the outcome obtained via the jackknife or K-fold cross-validation with benchmark dataset is actually from a combination of many different independent dataset tests. The combined parameter of AAC + RAAC + acACS was selected to identify the anticancer peptides in the independent dataset. The overall predictive accuracy (*Q*_*A*_) and Matthew’s correlation coefficient (*MCC*) in jackknife test were 89.33% and 0.787 by using of the SVM, respectively. This results showed that the new predictive method was not only able to achieve higher overall success rates, but also more stable.

### Evaluation of the predictive performances

In order to evaluate the predictive capability and reliability of the algorithm, the sensitivity (*S*_*n*_), specificity (*S*_*p*_), overall predictive accuracy (*Q*_*A*_) and Matthew’s correlation coefficients (*MCC*) are defined by


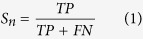



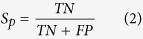



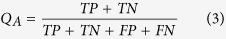



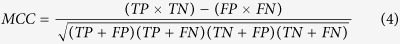


where *TP* denotes the numbers of the correctly recognized positives, *FN* denotes the numbers of the positives recognized as negatives, *FP* denotes the numbers of the negatives recognized as positives, *TN* denotes the numbers of correctly recognized negatives.

### Jackknife cross-validation test

In statistical prediction, the following three cross-validation methods are often used to examine a predictor for its effectiveness in practical application: independent dataset test, sub-sampling test, and jackknife cross-validation test^12^, among the three cross-validation methods, the jackknife test is deemed the most objective, and has been used to examine the performance of various predictors^13^,^14^,^15^,^16^. Hence the jackknife test was used to evaluate the performance of our method. During the process of jackknife test, each protein is singled out in turn for testing and the remaining proteins are merged for training.

## Discussion

Hajisharifi *et al*.^10^ used SVM-based classification on the base of their PseAAC parameter and the local alignment kernel as string kernel method in conjunction with 5-fold cross-validation test for the same benchmark dataset (see Equation (5)). For the purpose of comparing the predictive capability of our method, the predicted results of Hajisharifi’s and Chen’s method are enumerated in Table 2 for the same dataset. Compared results show that the performance of our method is more superior to that of Hajisharifi’s method. The *s*_*n*_, *s*_*p*_, *Q*_*A*_ and *MCC* of our method are about 5.40%,3.92%,4.49% and 0.095 higher than the predictive results of Hajisharifi’s method with 5-fold cross-validation test, respectively. Although only the *s*_*n*_ obtained in our method is higher than that of Chen’s method with 5-fold cross-validation test, the features of anticancer peptides are obtained more comprehensive in our method. At least our method can play a complimentary role to the existing methods in this area.

The predictive result indicates that the combined parameter AAC + RAAC + acACS is effective to the prediction of anticancer peptides proteins. From the discussion above, it can be seen that our method has advantage of more comprehensive features and higher predictive success rates. The combined parameter AAC + RAAC + acACS successfully enhance the prediction quality for the anticancer peptides. This method may have broad application in protein and DNA motif identification.

## Materials and Method

### Datasets

The Benchmark dataset was generated by Hajisharifi *et al*.^10^ and can be expressed as


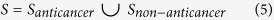


where *S*_*anticancer*_ consists of 138 anticancer peptides and *S*_*non-anticancer*_ consists of 206 non-anticancer peptides. The anticancer peptides derived from the antimicrobial peptides database (ADP2) and 206 non-anticancer peptides were selected from Universal Protein Resource^17^.

In order to estimate the effectiveness of the new prediction method, an independent dataset was employed and can be expressed as


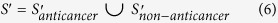


where 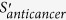 consists of 150 anticancer peptides and 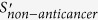 consists of 150 non-anticancer peptides. The samples in 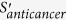 and 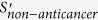 were fetched from the dataset used by Chen *et al*.^11^ and none of the sequences in *S*′ was the same with the sequences in *S*.

### Support Vector Machine (SVM)

The support vector machine (SVM) is a widely used classification method developed based on the statistical learning theory^18^,^19^,^20^,^21^,^22^,^23^,^24^,^25^. The SVM is particularly attractive to biological sequence analysis due to its ability to handle noise, large dataset and large input spaces. The SVM model is a representation of the examples as points in space, mapped by a kernel function so that the examples are divided by a clear gap that is widely enough. The new examples are mapped into the same space and predicted according to which side of the gap they fall on. The radial basis kernel function (RBF) was used to obtain the best classification hyperplane. The regularization parameter *C* and the kernel width parameter γ were tuned via the grid search method. For a brief formulation of SVM and how it works, see the papers^26^,^27^. In this paper, the LibSVM algorithm^28^ has been used to predict the anticancer peptide, which can be downloaded from http://www.csie.ntu.edu.tw/~cjlin/libsvm/.

### The local amino acids compositions (AAC)

The information parameters are very important for predicted algorithms. In a sequence-based predictor, the most important issue is the way in which to extract features from primary sequences of proteins^29^,^30^,^31^,^32^. The primary sequences of proteins are composed of 20 amino acids. The absolute occurrence frequencies of the 20 amino acids in protein are important features. Hence, the absolute occurrence frequencies of the 20 amino acids in protein sequence are considered as the information parameters of a protein and can be defined as


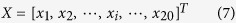


where 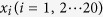 is the absolute occurrence frequencies of the 20 native amino acids and calculated by


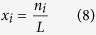


where *n*_*i*_ is the occurrence number of the 20 native amino acids of the protein; *L* is the length of the protein.

### Analysis of the amino acids compositions

**Figure 3:**
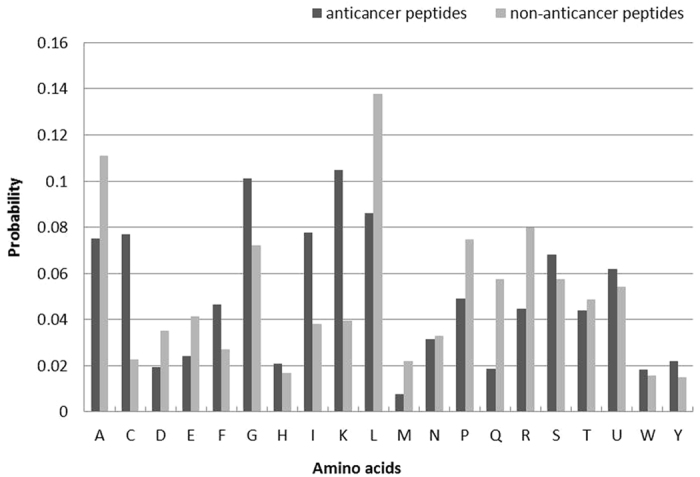
The different distribution of the amino acids compositions in anticancer peptides and non-anticancer peptides.

Using the dataset S, we analyzed the average amino acids compositions of anticancer peptides and non-anticancer peptides. The average amino acids compositions can be calculated as follow


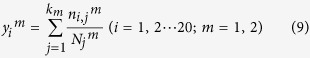


where 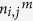 is the number of *i*-th amino acids of *j*-th protein in *m*-th group, 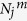 denotes the total number of amino acids of *j*-th protein in *m*-th group, 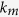 denotes the number of samples in the *m*-th group (here (*k*_1_ = 138, *k*_2_ = 206). We calculated the average amino acids compositions of anticancer peptides and non-anticancer peptides by using of Equation (9). The calculate results indicated that the amino acids compositions of anticancer peptides and non-anticancer peptides were different. Hence the amino acids compositions were suitable as features to distinguish anticancer peptides and non-anticancer peptides. The different distribution of the amino acids compositions in anticancer peptides and non-anticancer peptides were shown in Fig. 3.

### Auto covariance of the average chemical shift (acACS) algorithm

In a predictor, the most important issue is the way in which to extract features from primary sequences of protein. To achieve this, the acACS algorithm is proposed, which uses simple secondary structure information to represent the sample of a protein^33^. The average chemical shift of a protein has intrinsic correlation with the protein’s secondary structure and the function of this protein. According to this point of view, there must be some relationship among the average chemical shift, protein structure and functions. So the acACS algorithm has been widely applied to predictions of protein attributes, such as predicting protein submitochondrial localization^34^, the subcellular locations of the mycobacterial proteins and DNA-binding proteins^35^, as well as for identifying acidic and alkaline enzymes^36^ and discriminating between bioluminescent and nonbioluminescent proteins^37^. The acACS algorithm can be obtained from web server at http://202.207.14.87:8032/fuwu/acacs/index.asp.

For a protein *P*,


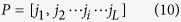


where *L* is the length of the protein sequence *p* and *j* represent the 20 native amino acids residues, *p* is then expressed as follow:


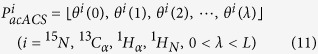


where θ^*i*^(*λ*) is the correlation factor of the average chemical shift for *j*_*l*_ with the average chemical shift for 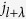 along the protein sequence. The factor 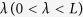 reflects the rank of correlation. The factor *i* can be the different composition of 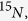 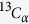, 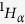 and 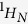. In order to obtain the best result, an appropriate number for factor *λ* and *i* should be determined according to the predicting results.

### The reduced amino acid composition (RAAC)

It was demonstrated that in the definition of global protein structure, the patterns of hydrophobic and hydrophilic residues have major significance. To obtain the hydropathy characteristics, the amino acids were divided into groups using their individual hydropathies according to the ranges of the hydropathy scale. Therefore, a protein sequence with 20 amino acids can be represented by a sequence with 6 characters according to following schemes: Strongly hydrophilic or polar (R, D, E, N, Q, K, H), Strongly hydrophobic (L, I, V, A, M, F), Weakly hydrophilic or weakly hydrophobic (S, T, Y, W), Proline (P), glycine (G) and Cysteine (C)^38^,^39^. The dipeptide composition of the six characters were chosen and represented as follow:


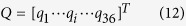


where 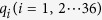 is the absolute occurrence frequencies of the 36 hydropathy dipeptides and calculated by


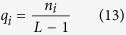


where 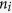 is the occurrence number of the 36 hydropathy dipeptides of the protein, *L* is the length of the protein.

## Additional Information

**How to cite this article**: Li, F.-M. and Wang, X.-Q. Identifying anticancer peptides by using improved hybrid compositions. *Sci. Rep*. **6**, 33910; doi: 10.1038/srep33910 (2016).

## Figures and Tables

**Table 1:** The predictive results of SVM by jackknife test for anticancer peptides.

Features	*s*_*n*_(%)	*s*_*p*_(%)	*Q*_*A*_(%)	*MCC*
AAC	87.68	94.66	91.86	0.830
RACC	77.54	88.35	84.01	0.642
acACS	71.02	90.29	82.56	0.633
AAC + RACC	86.96	97.57	93.31	0.861
AAC + acACS	86.23	97.57	93.02	0.856
RACC + acACS	77.54	91.74	86.05	0.707
AAC + RAAC + acACS	89.86	96.12	93.61	0.867

**Table 2:** The comparison of the predictive results between this paper and the other methods.

	Validation method	*s*_*n*_(%)	*s*_*p*_(%)	*Q*_*A*_(%)	*MCC*
This paper	Jackknife test	89.86	96.12	93.61	0.867
	5-fold cross-validation	90.58	96.60	94.19	0.879
Hajisharifi *et al*.^a^	5-fold cross-validation	85.18	92.68	89.70	0.784
iACP^b^	5-fold cross-validation	88.40	99.02	94.77	0.893

^a^See ref. 10.

^b^See ref. 11.
